# Supplementary figures and images for: Exploring the eating experience of a pneumatically-driven edible robot: Perception, taste, and texture
Source: PLoS One. 2024 Feb 5;19(2):e0296697. doi: 10.1371/journal.pone.0296697 (PMC10843494; doi:10.1371/journal.pone.0296697)

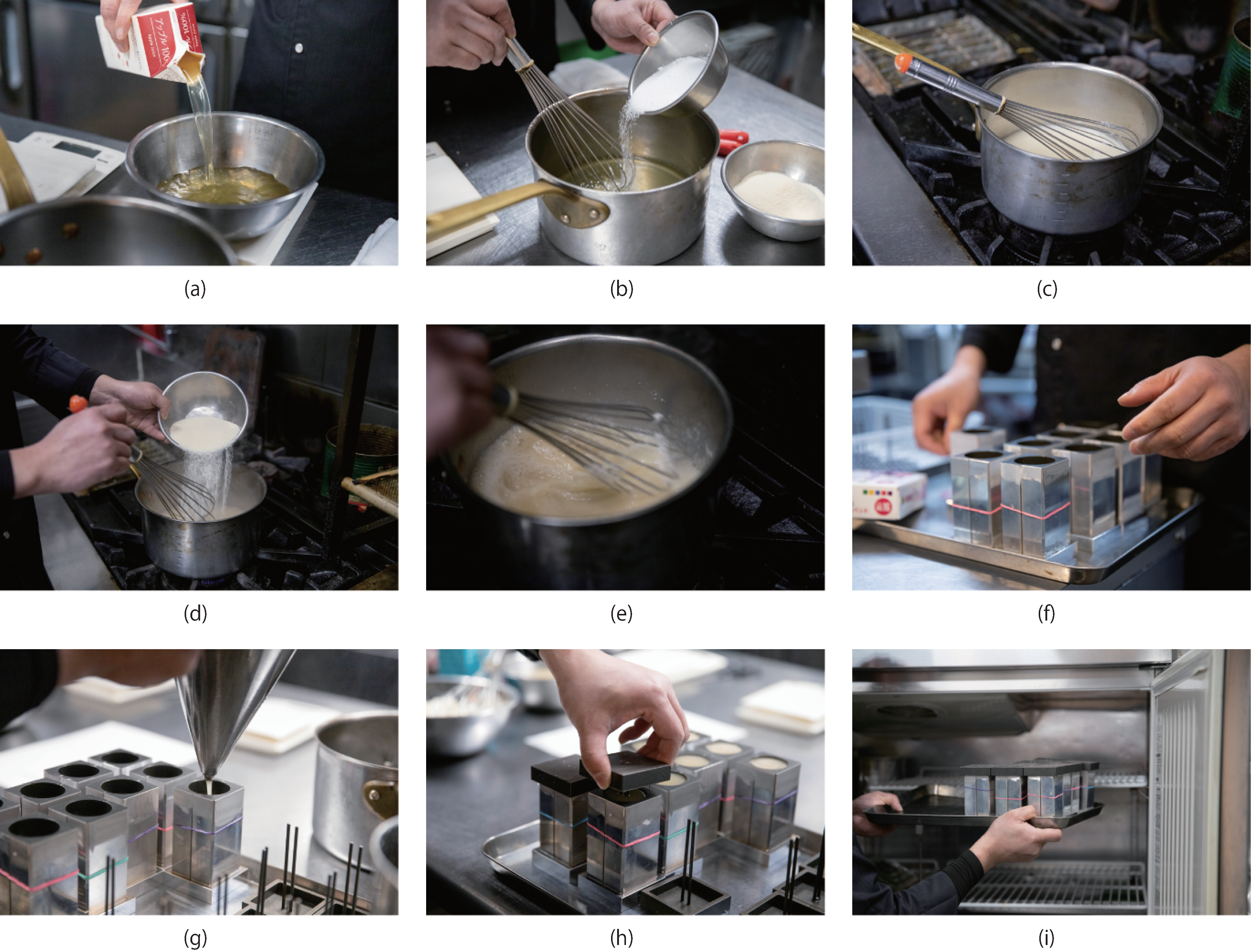

Supplement: S1 Fig — The process was performed in the order from (a) to (i). The edible parts were made in a kitchen and inspected by the Public Health Department. (a) Preparation of 100 mL of apple juice, (b) addition of 30 g of sugar and 1 g of calcium carbonate and mixing, (c) complete dilution of the sugar and calcium carbonate by heating, (d) addition of 25 g of gelatin, and (e) stirring until gelatin is completely dissolved. Removal from heat and allow slightly cooling. (f) During cooling, assemble the mold (fixing using a rubber band to prevent the mold from shifting). (g) Pouring of the mixed materials into the mold, (h) insertion of the mold to form the chambers of the edible part, and (i) refrigeration for half a day. (TIF) [file pone.0296697.s001.tif]
