# Supplementary material for: Exploring the eating experience of a pneumatically-driven edible robot: Perception, taste, and texture
Source: PLoS One. 2024 Feb 5;19(2):e0296697. doi: 10.1371/journal.pone.0296697 (PMC10843494; doi:10.1371/journal.pone.0296697)
Supplement: Attachment — Submitted filename: PLOS_ONE_Edible_Robot_Response_Letter.pdf [file pone.0296697.s002.pdf]

May 18, 2023

Dr. Santiago Casado Rojo
Guest Editor
*PLOS ONE*

Dear Editor and Reviewers:

We wish to resubmit the manuscript titled "Exploring the Eating Experience of a Pneumatically-Driven Edible Robot: Animateness, Guilt, and Texture Perception." The title has been updated in response to the reviewer's comment. The manuscript ID is PONE-D-23-00718.

We sincerely appreciate the time and effort you have dedicated to reviewing our work for publication in *PLOS ONE*. Your collective feedback has provided us with insightful and helpful comments. In response, we have made significant changes throughout the manuscript, including an edited title and references to address the reviewers' concerns. We believe these revisions strengthen our paper's conclusions, and it is with great pleasure that we resubmit our manuscript for further consideration. We hope our corrections and responses adequately address the concerns raised during the review process, and we eagerly await your feedback on this submission. In the following response, we have included our detailed replies to all comments from the reviewers (reviewer comments are inserted in text boxes). Revised sentences in the manuscript are highlighted in red color. Additionally, a highlighted revised manuscript is included. Finally, we would like to note that we have sought professional editing services to proofread and polish the English language of our manuscript prior to resubmission.

Thank you for your consideration. I look forward to hearing from you.

Sincerely,

Yoshihiro NAKATA
Graduate School of Informatics and Engineering,
The University of Electro-Communications
1-5-1, Chofugaoka, Chofu, Tokyo 182-8585 Japan
E-mail: ynakata@uec.ac.jp

# Response to Reviewer 1

**General comment**

This paper explores the intriguing experience of eating robots. It evaluates the psychological impact of eating a ”leaving” creature. The authors designed an experience that involves two aspects that are not regularly experienced together: watching the creature move (i.e. being alive), and eating it. I find this idea highly interesting and I believe it has a very strong contribution to a wide range of research fields ranging from human-robot interaction to understanding human behavior.

The overall presentation of the paper is good. It is well-written and the arguments are clearly presented. The description of the robot's fabrication process is detailed and clear.

While the paper is highly innovative and interesting, some aspects of the work should be further explained.

**Answer**

Thanks to the reviewer's valuable comments. Detailed responses to all comments are listed below.

**Comment 1**

I believe that the main contribution of this paper is in evaluating the experience of eating a robot. As such, the design considerations that contribute to the experience should be elaborated. The specific robot appearance and the movements chosen for the first experiment should be justified and an explanation of the automatic social experience triggered when interacting with non-humanoid robots should be presented in order to strengthen the claim that there is a significant difference between eating a moving vs not moving robots (see suggested references at the end of the review).

**Answer 1**

As the reviewer has pointed out, the primary contribution of this paper is the evaluation of the unprecedented experience of eating a robot. As (to our knowledge) this was the first experiment conducted to evaluate the experience of eating a robot, we opted to use a robot with the simplest possible geometry. Additionally, the robot's design was constrained by two conditions:

1. Firstly, the robot had to be placed in the mouth. We wanted the participants to experience eating a moving robot rather than merely consuming it. Therefore, it was not feasible to cut the robot into smaller pieces before eating. It was necessary to design a shape and size that would enable the robot to be placed directly into the mouth. Thus, we designed the robot in the shape of a stick.

2. Secondly, the robot had to be pneumatically driven. We chose air as the power source for the robot because it is safe to use in the mouth. Due to the robot's size, we were limited to two air chambers inside it. For our experiments, we utilized two types of movements (sideway swinging and vertical stretching) as stimuli, which could be achieved by controlling the air supply to these two air chambers.

Some sentences have been added to the "Structure" section regarding the shape of the robot we designed. Specifically,

> 2nd paragraph, Structure, Edible robot (page 3, line 76)
"Fig. 1 shows the three-dimensional (3-D) computer-aided design model of the edible robot and its internal structure. It consists of an edible part made of material described in the "Edible material"

section, and a base component, which is connected to an air tube to supply air to the edible part. As this was the first experiment that aimed to allow participants to experience eating a moving rather than a motionless robot, it was not feasible to cut the robot into smaller pieces before eating. Consequently, we had to design a shape and size that would enable the robot to be placed directly into the mouth; this led to stick-shaped robots. Additionally, owing to the constraint of the size of the robot that could fit in the mouth, the edible part was designed to have only two air chambers."

Thank you for suggesting related papers on the "automatic social experience triggered when interacting with non-humanoid robots." It is plausible that the movement of the robot may have provided social cues to the participants, whether we intended it or not. The observation of the robot's movement before eating it may have influenced the results. In Experiment 2, the robot was placed in the mouth for a specific period to impress the experience of eating a moving robot. However, the observation of the robot before eating should be taken into consideration. We have included a description of this limitation in the "Discussion" section.

> 3rd paragraph, Discussion (page 17, line 504)
"We designed the color and appearance of the robot as simple and featureless because we did not want those factors to affect the evaluation. In the case the experience of eating a living creature is intended to be realized using the edible robot, it will be necessary to make the robot look more like a living creature. Furthermore, the motion of the robot could trigger the participant's social experience [26–28] and influence the results. Thus, the motion should be carefully designed. Another option would be to blindfold the participant so that the robot's appearance does not affect the evaluation during the experiment."

[26] Anderson-Bashan L, Megidish B, Erel H, Wald I, Hoffman G, Zuckerman O, et al. The greeting machine: An abstract robotic object for opening encounters. In: 2018 27th IEEE International Symposium on Robot and Human Interactive Communication (RO-MAN); 2018. p. 595–602.
[27] Erel H, Shem Tov T, Kessler Y, Zuckerman O. Robots are always social: Robotic movements are automatically interpreted as social cues. In: Extended Abstracts of the 2019 CHI Conference on Human Factors in Computing Systems. CHI EA '19. New York, NY, USA: Association for Computing Machinery; 2019. p. 1–6.
[28] Ju W, Takayama L. Approachability: How people interpret automatic door movement as gesture. International Journal of Design. 2009;3(2):77–86.

**Comment 2**

It is important to explain what was the predicted experience for each of the six videos and to translate the technical variables into experience terms. What did the authors expect participants to experience and why the specific technical manipulation of the object is predicted to lead to such experience.

**Answer 2**

The six videos were designed to showcase the six different movements achieved based on the combination of two motions (lateral swinging and vertical stretching), which can be manipulated by varying the timing of air supply to the two air chambers inside the edible part, as well as three levels of speed achieved by adjusting the cycle of air supply and exhaust. The lateral swinging motion produces movements similar to spinal flexion, while the vertical stretching motion produces movements similar to the upward and downward motions of the torso caused by knee flexion and extension. We considered that the speed of the movements could express the difference in alertness where fast movements can appear

exciting, and slow movements can appear relaxed. Although it cannot be claimed that faster movements are more lifelike, we expected that movements that are too slow would compromise the robot's lifelikeness. We have included additional explanations in the Section on Experiment 1, as follows:

> 1st paragraph, Robot movement conditions, Experiment 1 (page 7, line 232)
"Two factors were manipulated to control movement of the developed edible robot: the timing and cycle of air supply and exhaust to and from the two air chambers in the edible part. For the timing of the air supply and exhaust, we set up two conditions: the first was one air chamber at a time (hereafter, referred to as the "alternating" condition), and the second condition was the cycle of air supply and exhaust in which air was supplied to both air chambers (hereafter referred to as the "simultaneous" condition). In the alternating condition, the robot swings laterally, and the motion produces movements similar to spinal flexion. In contrast, in the simultaneous condition, the robot moves up-and-down, and the motion produces movements similar to the up-and-down motion of the torso caused by knee flexion and extension. In addition, for the air supply and exhaust cycle, we set up three conditions: short, middle, and long (see Table 4 for each condition). We considered that the speed of the movements could express the difference in alertness, where fast movements can appear exciting, and slow movements can appear relaxed. Although it cannot be claimed that faster movements are more lifelike, we expected that movements that are too slow would compromise the robot's lifelikeness. Six conditions were set up as described above, and the participants participated in all conditions."

**Comment 3**

In experiment 1 The use of the "object" instead of "robot" is appreciated. However, the authors should explain if the order of the videos was counterbalanced and if they controlled for order effects.

**Answer 3**

Thank you very much for your comments. We apologize for the oversight and appreciate your suggestion. We indeed counterbalanced the order of the videos and controlled for order effects, but failed to mention it in the manuscript. We have now updated the text as follows:

> 1st paragraph, Procedure, Experiment 1 (page 9, line 272)
"After agreeing to participate in Experiment 1, participants were presented with a 15-second video showing the movements of the object (the edible robot). They then answered 10 questions regarding their impression of the object's movements. Participants watched the videos of all six conditions. The order of these videos was randomized across participants to control for order effects."

**Comment 4**

It is important to explain why the authors asked about the perceived taste in their survey (experiment 1). These questions may bias the perception of the robot's animacy which was the main purpose of this study

**Answer 4**

Thank you for your comment. We apologize for not providing a clear explanation for the inclusion of perceived taste in our survey. The purpose of Experiment 1 was the conduct a preliminary investigation to ascertain whether changes in the movement of an object (representing food) would result in a

corresponding change in appetite perception. The results suggest that participants may be influenced by appetite even by watching a moving object. Based on these results, a more detailed taste evaluation (sweet, hot, sour, bitter, and spicy) was included as a criterion in Experiment 2, which involved actual food consumption. To clarify this issue, we revised the methodology Section of Experiment 1, as it was not adequately explained previously.

> 1st paragraph, Question items, Experiment 1 (page 8, line 250)
"To investigate animacy toward the edible robot, participants were asked to respond to scales such as animateness or emotion. In addition, additional questions were added regarding the taste and morality to investigate whether the emotions toward eating the robot, such as appetite and morals, change just by watching the movements of the edible robot. For each of the six videos, the participants were asked to respond to the following eight items based on a 7-point Likert scale. The following are the questionnaire items."

**Comment 5**

It would be better to report the results of Experiment 1 immediately after its method.

**Answer 5**

Thank you very much for your valuable suggestions. We agree that reporting the results immediately after the methods for each experiment improves readability. We have now restructured the paper by establishing two separate sections, "Experiment 1" and "Experiment 2". The results are now displayed immediately after the description of the methods pertaining to Experiment 1, followed by the method and results of Experiment 2. The revised manuscript is organized as follows:

Introduction
Edible robot
- Structure
- Operating principle
- Edible material
- Tensile-strength testing
- Hardness measurements
- Robot development
- Control system

Experiment 1
- Participants
- Robot movement conditions
- Question items
- Procedure
- Ethical considerations
- Results

Experiment 2
- Participants
- Robot movement conditions
- Question items
- Procedure
- Ethical considerations
- Results

Discussion
Conclusion

**Comment 6**

In experiment 1, it is not clear why each question was analyzed separately. It seems that the questions can be divided into two categories - the perception of the robot as animated and the perception of the robot as food - while it would be better to test the validity and reliability of these two categories, I acknowledge that it is beyond the scope of this paper, but I think each category should be analyzed as a subscale instead of analyzing the answer to each question separately. This is in line with the conclusions that the authors derive for experiment 1 (separately discussing animacy and taste).

**Answer 6**

Thank you for your comment. We appreciate your suggestion to analyze the questions as subscales based on the two categories of perception of animacy and taste. The reason for analyzing perception and taste independently in Experiment 1 was to conduct an exploratory and preliminary study to determine if changes in taste perception occur simply by watching the robot move. However, the actual statistical results also suggest that the two are independent.

First, we performed a factor analysis of the results of the questionnaire survey in Experiment 1. Based on the scree plot (see Fig. A), we determined that two factors were appropriate. The factor loadings for these two factors indicated that perception (animateness, emotion, and intelligence) and taste (Do you think it looks tasty? Do you want to eat it?) were distinct factors (see Table A). Furthermore, in Experiment 2, we conducted a correlation analysis of the relationship between perception and taste for each question. The results showed no significant relationship between perception and taste. These findings suggest that perception and taste are indeed independent of each other.

Although we do not plan to revise the manuscript based on this suggestion, we believe that this explanation addresses your concern.

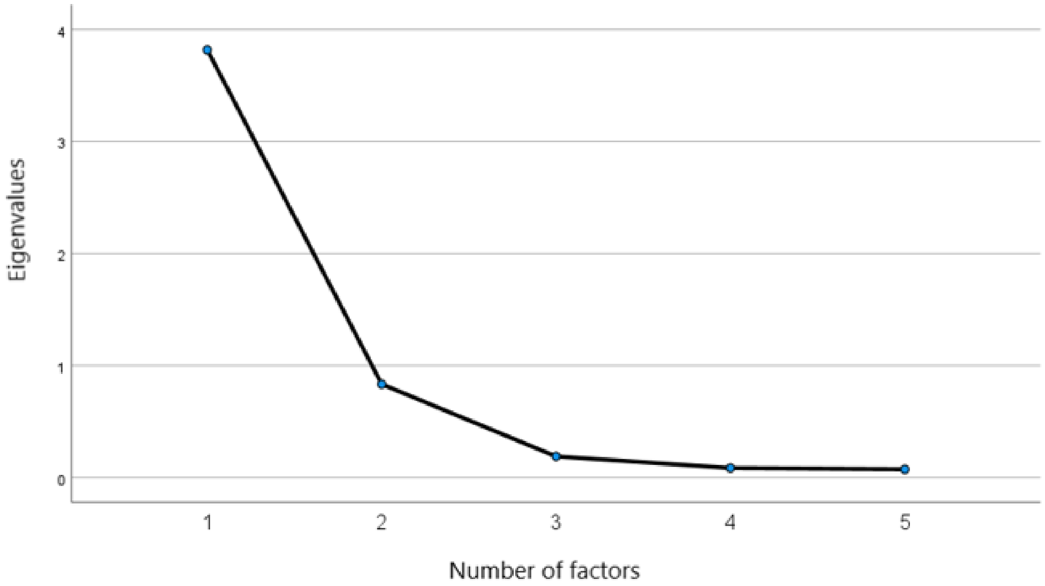


**Fig A. Scree plot**

**Table A. Pattern matrix**

| | Factor | |
|---|---|---|
| | 1 | 2 |
| Emotion | 1.023 | -0.045 |
| Animateness | 0.880 | 0.040 |
| Intelligence | 0.846 | 0.079 |
| Do you think it looks tasty? | -0.017 | 1.010 |
| Do you want to eat it? | 0.063 | 0.879 |

Factor extraction method: Maximum Likelihood Method
Rotation method: Promax method with Kaiser's normalization

**Comment 7**

The authors should also explain how they analyzed the answers to the open questions (thematic analysis?) and if possible provide the percentage of participants that gave the answers reported in the paper.

**Answer 7**

Thank you for your comment. Regarding the analysis of the open-ended questions, we initially conducted a frequency analysis of the words that were present and did not perform a thematic analysis. However, we appreciate your comment; in response, we have included tables in "Results" section in Experiment 1 and "Results" section in Experiment 2 that present the top 5 answers for each open-ended question.

> 5th paragraph, Results, Experiment 1 (page 10, line 324)

"In addition, Tables 6 and 7 show the results of the responses to the questions "What impression did you have of the object's movements?" and "What do you think it tastes like?" These tables list the top five words for each of the simultaneous and alternating conditions. In the simultaneous condition, participants perceived the robot as hard, whereas in the alternating condition, participants perceived it as soft. Additionally, in the simultaneous condition, participants perceived the robot as tasteless, whereas in the alternating condition, participants perceived it as sweet-tasting."

> Table 6 and 7, Experiment 1 (page 12)

**Table 6. Free-form descriptions: What impression did you have of the object's movements?**

| Simultaneous | Alternating |
|---|---|
| Hard | Soft |
| Impression | Disgusting |
| Movement | Intense |
| Shaking | Not particularly |
| Stiff | Pulling |

Note: List of the top five words for each condition.

**Table 7. Free-form descriptions: What do you think it tastes like?**

| Simultaneous | Alternating |
|---|---|
| Tasteless | Sweet |
| Orange | Jelly |
| Bitter | Delicious |
| Pasta | Gummy candy |
| Salty | Apple |

Note: List of the top five words for each condition.

> 2nd paragraph, Results, Experiment 2 (page 14, line 421)
"In addition, Tables 8, 9, and 10 show the results of the responses to the questions "What was your impression of the food you just ate?" "What did it taste or smell like?" and "What food ingredients do you think the food you just ate is made of?" These tables list the top five words for each of the stationary and movement conditions. In the stationary condition, participants perceived the robot as a "food", whereas in the movement condition, participants perceived the robot as a "creature". These results also indicate that the edible robots were able to make the participants feel that they were eating animated beings in the movement condition. Additionally, in the evaluation of the taste of the robot, participants frequently described it as "juice" and "fruit" in the stationary condition; by contrast, in the movement condition, they frequently described it as "sweet" and mentioned specific tastes such as "apple." Moreover, in the stationary condition, participants thought the robot was made of "gelatin" and "sugar", but in the movement condition, participants thought the robot was made of "agar" and "fruit juice". In this way, participants thought the robot was made of different materials under different conditions."

> Table 8, 9, and 10, Experiment 2 (page 14–15)

**Table 8. Free-form descriptions: What was your impression of the food you just ate?**

| Stationary | Movement |
|---|---|
| Eating | Movement |
| None in particular | Creature |
| Food | Mouth |
| Jelly | Disgusting |
| Delicious | Bite |

Note: List of the top five words for each condition.

**Table 9. Free-form descriptions: What did it taste like or smell like?**

| Stationary | Movement |
|---|---|
| Smell | Sweet |
| Juice | None in particular |
| Eat | Apple |
| Fruit | Juice |
| Sweets | Flavor |

Note: List of the top five words for each condition.

**Table 10. Free-form descriptions: What food ingredients do you think the food you just ate is made of?**

| Stationary | Movement |
|---|---|
| Gelatin | Agar |
| Sugar | Fruit juice |
| Water | Sweetness |
| Apple | Artificial |
| Juice | Taste |

Note: List of the top five words for each condition.

**Comment 8**

Some statements should be toned down (e,g, "a robot that moves like a living creature")

**Answer 8**

Thank you for pointing this out. We acknowledge that some of our statements were indeed exaggerated. We have carefully reviewed the entire manuscript and toned down the following sentences:

> 1st paragraph, Experiment 1 (page 7, line 220)

"The purposes of this study were to fabricate an edible robot system that artificially provides the biological characteristics to food, and to examine the effects of robot movement on the eating experience. In Experiment 1, an online survey was conducted to determine which movements aroused feelings of "animateness, appetite, and morality" toward robots which were eaten by presenting various robot movements to the participants."

> 1st paragraph, Question items, Experiment 1 (page 8, line 250)

"To investigate animacy toward the edible robot, participants were asked to respond to scales such as animateness or emotion. In addition, additional questions were added regarding the taste and morality to investigate whether the emotions toward eating the robot, such as appetite and morals, change just by watching the movements of the edible robot. For each of the six videos, the participants were asked to respond to the following eight items based on a seven-point Likert scale."

> 1st paragraph, Experiment 2 (page 12, line 332)

"Using the movements identified in Experiment 1, we investigated in Experiment 2 the types of impressions participants had when they actually ate a robot that moved."
NOTE: We have deleted "like a living creature."

> 1st paragraph, Question items, Experiment 2 (page 12, line 343)

"To examine whether emotions such as "animateness," "appetite," and "guilt" for eating the edible robot changed in response to the robot's movements, the participants were asked to answer the following thirteen Likert-scale question items for all stationary and movement conditions."

> 1st paragraph, Question items, Experiment 2 (page 12, line 348)

"In Experiment 1, we used the index of "morality," but in Experiment 2, we revised it to "guilt," a question item that is more likely to be linked to a specific action, to evaluate the impression of eating a robot."

> 4th paragraph, Results, Experiment 2 (page 16, line 450)

"Moreover, to achieve the objective of this study, i.e., to examine whether participants feel guilt when they eat the edible robot, a Kendall rank correlation coefficient analysis was conducted between the results associated with "the object has a feeling of animateness" and "feel guilty" outcomes."

> 1st paragraph, Discussion (page 16, line 462)
"This study conducted an experiment in which an edible robot was developed using gelatin and sugar to assess human impressions by allowing them to eat its moving parts."
NOTE: We have deleted "when eating live creatures."

**Comment 9**

The low number of participants in Experiment 2 should be discussed as a limitation as well as the choice of a within-participants experimental design. Such a within experimental design is subjected to order effects (counterbalancing the conditions does not resolve this since after experiencing a robot that moves, a robot that doesn't move may be perceived as "dead" instead of as an object). This choice should be explained and should be clearly discussed as a limitation of the work.

**Answer 9**

Thank you for your constructive comments. As you have noted, given the implementation of a within-participant design in Experiment 2, it is important to address the issue of order effects as a potential limitation. Accordingly, we conducted an analysis of order effects. In each of the stationary and movement conditions, we compared the group that received the stationary condition first to the group that received the movement condition first to see if each measure was affected by the order effect. No significant differences were found between the groups. However, we believe that this is an important point, so we have added it to the results of Experiment 2 and revised the manuscript accordingly.

> 1st paragraph, Results, Experiment 2 (page 14, line 415)
"As this study was based on a within-participant design, we checked for order effects; no significant effects of the order of conditions were identified. In addition, no significant correlations were found between the items measuring participants' perception of the robot (animateness, emotion, and intelligence) and those measuring taste (tasted good, sweetness, sourness, bitterness, spiciness, umami, and saltiness)."

> 3rd paragraph, Discussion (page 17, line 511)
"In Experiment 2, wherein the participants actually ate the food, we conducted within-participant comparisons because of time constraints. However, we believe that in the future it would be better to conduct inter-participant comparisons to offset the order effect. In addition, we believe that the use of semistructured interviews as well as questionnaires, will enable us to gain a deeper understanding of participants' impressions of the edible robots."

**Comment 10**

Another choice that should be clarified is the use of onomatopoeia as a measure. Why is it the best fit? Is it a validated measure? What is its contribution over other well-validated measures?

**Answer 10**

We appreciate your insightful comments. In response to your concerns about the use of onomatopoeia as a measure, we would like to clarify our rationale for selecting this approach. Previous studies have demonstrated that onomatopoeia is particularly effective in expressing qualitative impressions that are difficult to quantify numerically, such as the sensation of symptoms of illness [23] and

impressions of food [24]. For this reason, we decided to use this scale in our study. We have now added a comment on this issue in the revised text.

> 2nd paragraph, Question items, Experiment 2 (page 13, line 364)
"In addition to the thirteen Likert-scale questions, fourteen onomatopoeia related to eating (e.g., Gabu (Grappling), Kori–Kori (Crisp)) were extracted from the Japanese Onomatopoeia Dictionary [22]. Participants were asked to respond to the texture of what they just ate with the corresponding onomatopoeia (multiple responses allowed). Onomatopoeia is said to be effective in expressing sensations that are difficult to quantify, such as texture. For example, onomatopoeia has been reported to be effective in conveying the sensation of symptoms of illness [23]. Onomatopoeia is also said to be effective in conveying food impressions [24]. Unlike scales that ask for impressions with numerical values, onomatopoeia is superior in that it can express differences in quality that cannot be captured with mere numerical values."

[23] Doizaki R, Matsuda T, Utsumi A, Sakamoto M. Constructing a system which proposes metaphors corresponding to the onomatopoeia expressing medical conditions. International Journal of Affective Engineering. 2016;15(2):37–43.
[24] Kato A, Fukazawa Y, Mori T. Discovering pattern of onomatopoeia used in food reviews. In: The 6th International Conference on Soft Computing and Intelligent Systems, and The 13th International Symposium on Advanced Intelligence Systems; 2012. p. 502–507.

**Comment 11**

I am also wondering why the authors did not conduct an in-depth semi-structured interview. With 16 participants and such an intriguing innovative research question, it would be highly informative to get details about the participants' subjective experience (if analyzed appropriately).

**Answer 11**

Thank you for your comment. As you have noted, it would have been desirable to conduct semistructured interviews to gain a deeper understanding of their impressions about eating. However, due to time constraints within the scope of the experiment, it was not possible to conduct these interviews. As an alternative, free-form descriptions were utilized to supplement this limitation. We recognize the importance of this point and have taken note to include it as a limitation in the "Discussion" section.

> 3rd paragraph, Discussion (page 17, line 511)
"In Experiment 2, wherein the participants actually ate the food, we conducted within-participant comparisons because of time constraints. However, we believe that in the future it would be better to conduct inter-participant comparisons to offset the order effect. In addition, we believe that the use of semistructured interviews as well as questionnaires, will enable us to gain a deeper understanding of participants' impressions of the edible robots."

**Comment 12**

Some objective measures would also benefit such a novel exploration (e.g. reaction time measures or coding participants' behavior).

**Answer 12**

Thank you for your valuable suggestion. Unfortunately, in this study, we did not conduct objective evaluations in this experiment. However, we completely agree that incorporating objective measures would be advantageous for such a novel study. For example, in Experiment 2, instead of relying solely on subjective questionnaires, we could have assessed participants' guilt or hesitation about eating the robot by measuring the time it took for them to actually bite and eat it after being instructed to do so. As the robots are powered by air pressure, when bitten, the air pressure inside the air chamber decreases; this change could be used to measure the time to bite. We would like to consider incorporating such objective evaluations based on behavior in future research. We have added comments on the lack of objective evaluations as limitations in the Discussion as follows:

> 4th paragraph, Discussion (page 17, line 517)
"In this study, we recognize the limitation of not incorporating objective evaluations, which could have provided additional insights into participants' guilt or hesitation about eating the robot. For instance, in Experiment 2, measuring the time it took for participants to bite and eat the robot after they were instructed to do so could have served as an objective measure that could complement the subjective questionnaires. As the robots are powered by air pressure, the decrease in air pressure inside the air chamber upon biting could be utilized to measure the time to bite. In future research, we aim to address this limitation by incorporating objective evaluations based on behavior, thus enhancing further the validity of our findings."

We acknowledge that the lack of objective measures, such as the time it takes for participants to bite and eat the robot, is a limitation of our study that we will address in future research.

**Comment 13**

The authors should also clarify why participants were asked to hold the object in their mouth before eating it. This is not clear and may bias the experience (from a regular eating experience).

**Answer 13**

In our study, the aim was to investigate the impact on people's perception when they eat the robot while it is still moving, rather than eating it after it has stopped moving. The edible robot moves by supplying air to the air chamber, which expands, causing the robot to move. Once the robot is bitten and the air chamber is punctured, it can no longer move. To ensure that participants properly recognized the sensation of the moving robot in their mouth and to accurately evaluate the difference in impressions between the moving and non-moving conditions, we instructed them to hold the robot in their mouths for a certain period of time before eating it. A relevant explanatory comment has been added, as indicated below:

> 1st paragraph, Procedure, Experiment 2 (page 13, line 380)
"After the participants agreed to participate in the experiment in the front chamber, they were moved to a laboratory where an edible robot was placed on a desk. After 15 seconds, the participants held the 3-D printed handle and lifted the edible robot with their right hands and kept the edible part in their mouths for 10 seconds. The reason for keeping the robot in their mouths for 10 seconds was to

ensure that participants fully recognized the sensation of the robot moving inside their mouths under the moving condition."

**Comment 14**

The chi-square analysis should be better explained. What were the variables included (not conditions since there is a separate analysis for each condition) and what is onomatopoeia type? This was not defined in the method section.

**Answer 14**

Thank you for your feedback and suggestions. We apologize for the lack of clarity in our previous explanation and appreciate your comments on the need for further information on the variables included and the definition of "onomatopoeia type." Moreover, we regret an oversight in our methodology. Rather than the chi-square test, we realized that the Cochran's Q test would be more appropriate for our experiment since the participants were allowed to choose multiple onomatopoeia. Therefore, we have revised our manuscript accordingly.

To address your feedback, we have added a new table (Table 11) to the 'Results' section in Experiment 2, which presents the frequency of different types of onomatopoeia mentioned by the participants. This table was used to conduct the Cochran's Q test, and we hope that it will help clarify our analysis. The manuscript has been updated as follows:

> 3rd paragraph, Results, Experiment 2 (page 15, line 436)
"Subsequently, we examined whether the texture of the edible robot when chewed differed between the tested conditions. For texture, participants were asked to multiple select the corresponding onomatopoeia. Table  lists the onomatopoeia selected by the participants in each condition. In the stationary condition, there was a significant deference on the onomatopoeia type (Cochran's $Q$ (13) = 42.23, $p$ < 001). Adjusted multiple comparisons indicated that "Munya–Munya (mumble)" was selected significantly more often than Musha–Musha (Munching), Picha–Picha (Splashing), Bori–Bori (Munching), Gatsu–Gatsu (Gobbling), Gari–Gari (Crunchy), Kari–Kari (Crunchy) and Kusha–Kusha (Crunchy). On the other hands, in the movement condition, there was a significant deference on the onomatopoeia type (Cochran's $Q$ (13) = 48.07, $p$ < 001). Adjusted multiple comparisons indicated that "Kori–Kori (Crisp)" and "Gabu (Grappling)" were selected significant trend of more often than Bori–Bori (Munching), Gatsu–Gatsu (Gobbling), Gari–Gari (Crunchy), Kari–Kari (Crunchy) and Kusha–Kusha (Crunchy)."

We believe that these changes address your concerns and improve the clarity of our paper. In the case these are not sufficient, we would gladly revisit these listings and revise accordingly based on additional reviewer comments. Once again, we appreciate your feedback and apologize for any confusion caused by our inadequate description.

**Table 11. Number of onomatopoeia that appeared for each condition**

| Onomatopoeia | | Condition | |
|---|---|---|---|
| Japanese | English | Stationary | Movement |
| Kori–Kori | Crisp | 7 | 8 |
| Gabu | Grappling | 4 | 8 |
| Munya–Munya | Mumble | 8 | 1 |
| Nicha–Nicha | Sticky | 3 | 7 |
| Guchu | Sopping | 4 | 6 |
| Kucha–Kucha | Crunching | 4 | 6 |
| Gappuri | Firmly (grasped) | 3 | 4 |
| Musha–Musha | Munching | 1 | 2 |
| Picha–Picha | Splashing | 1 | 1 |
| Bori–Bori | Munching | 0 | 1 |
| Gatsu–Gatsu | Gobbling | 0 | 0 |
| Gari–Gari | Crunchy | 0 | 0 |
| Kari–Kari | Crunchy | 0 | 0 |
| Kusha–Kusha | Crunchy | 0 | 0 |

**Comment 15**

The authors should report exact p values and 0.1 cannot be considered marginally significant.

**Answer 15**

Thank you for pointing this out. We apologize for the inadequate description of the statistical analysis. We intended to convey the notion that there was a "significant trend," and we appreciate your suggestion to clarify this. To avoid any misunderstandings that may arise among readers due to the inappropriate description, we have replaced the phrase "marginally significant" with "significant trend" in the "Results" section in Experiment 2, thus emphasizing that our findings exhibit a meaningful (yet statistically insignificant) pattern.

> 1st paragraph, Results, Experiment 2 (page 14, line 404)
"Because the normality of the data could not be verified, Wilcoxon signed-rank tests were performed on the 13 Likert scale questions for comparisons of stationary and movement conditions. Given the thirteen separate Wilcoxon signed-rank tests, an alpha value was set and used to control for type-I errors. The results either indicated that four items, i.e., the "animateness (W = 134.50, z = 3.46, $p < 0.05$, r = 0.87), emotion (W = 64.50, z = 2.81, $p < 0.10$, r = 0.70), intelligence (W = 66.00, z = 2.97, $p < 0.10$, r = 0.74), and guilt (W = 88.50, z = 3.07, $p < 0.05$, r = 0.77)" for the edible robot were significantly higher or significant trends were yielded in the movement compared with the stationary condition."

> 3rd paragraph, Results, Experiment 2 (page 15, line 439)
"In the stationary condition, there was a significant deference on the onomatopoeia type (Cochran's $Q$ (13) = 42.23, $p <$ 001). Adjusted multiple comparisons indicated that "Munya–Munya (mumble)" was selected significantly more often than Musha–Musha (Munching), Picha–Picha (Splashing), Bori–Bori (Munching), Gatsu–Gatsu (Gobbling), Gari–Gari (Crunchy), Kari–Kari (Crunchy) and Kusha–Kusha (Crunchy). On the other hands, in the movement condition, there was a significant deference on the onomatopoeia type (Cochran's $Q$ (13) = 48.07, $p <$ 001). Adjusted multiple comparisons indicated that "Kori–Kori (Crisp)" and "Gabu (Grappling)" were selected significant trend of more often than Bori–Bori (Munching), Gatsu–Gatsu (Gobbling), Gari–Gari (Crunchy), Kari–Kari (Crunchy) and Kusha–Kusha (Crunchy)."

**Comment 16**

Comments 5-7 about the analysis of Likert and open questions are also relevant to experiment 2.

**Answer 16**

Thank you for your comment. As you have pointed out, Comments 5–7 are indeed also relevant to Experiment 2. In Experiment 2, we conducted correlation tests for each condition (without and with movement) and found no significant correlations between "animateness, emotion, and intelligence" and "taste" (tasted good, sweetness, sourness, bitterness, spiciness, umami, and saltiness). In other words, we confirmed that taste was not correlated with perception factors. Therefore, we believe that it is reasonable to analyze perception and taste separately. We consider this point to be important. Accordingly, we have incorporated the correlation results to the results of Experiment 2 in the revised text.

> 1st paragraph, Results, Experiment 2 (page 14, line 415)

"As this study was based on a within-participant design, we checked for order effects; no significant effects of the order of conditions were identified. In addition, no significant correlations were found between the items measuring participants' perception of the robot (animateness, emotion, and intelligence) and those measuring taste (tasted good, sweetness, sourness, bitterness, spiciness, umami, and saltiness)."

**Comment 17**

In the discussion the authors should discuss the wide range of implications their study has for the research community, both in terms of psychological experiences and for the HRI community. What can we learn about human nature and eating habits? How does reminding people that their food is/was alive changes their eating experience? For the HRI community, there are great implications for understanding the concept of non-humanoid robots and their perception as leaving creatures. If people feel guilty eating food that has autonomous movement this highlights humans' tendency to animate very simple objects that perform a minimal movement - to the extent that they feel guilty when eating them despite clearly understanding that they are not alive.

**Answer 17**

Thank you for your very important and thought-provoking comments. We have added a comment to describe how the research on eatable robots can contribute to the surrounding area and the HRI community, based on the comments we have received.

> 6th, 7th, and 8th paragraphs, Discussion (page 17, line 538)

"Finally, we believe that the appeal of research on eating-enabled robots lies in the fact that it stimulates discussion in various interdisciplinary research areas. For example, academic contacts with the humanities are very interesting. In many cases, we humans prekill food creatures and then cook and eat them. Nowadays, the presence of these living animals is greatly reduced when food arrives at the table. However, in essence, we humans are also animals, as many animals feed themselves by eating living creatures. In other words, our eating behavior is a combination of cultural, social, and animal factors. In particular, it is useful to consider the animal component of human-eating behavior, for

example, how our appetites are aroused by moving entities in the outside world, and how this leads to eating behaviors. From this biological point of view, we believe that the edible robot we have developed, which can control its movements and sense of taste, has great potential for the study of eating behavior. Furthermore, our robot may also be useful in cultural anthropology to examine how such biological human elements are combined with cultural elements to create a food culture and identify the minimalistic elements of this culture. As described above, our robot is expected to be an effective tool for the integrated exploration of the humanities and social sciences, biology, and psychology pertaining to eating behaviors.

The development of a robot that gives the impression of being alive, and the experience of eating it has the potential to lead to food education. We expect that the broadening of philosophical discussions on topics such as what life is and the emotions that arise when we eat robots, as these are modulated by the robot we have developed on this study, will lead to a deeper understanding of bioethics and will stimulate moral education efforts.

In conventional studies of human–robot interactions, the impression of the robot was often ascertained by participants in experiments in the form of questionnaire ratings. These subjective methods often failed to reflect unconscious human processes. We believe that giving users the experience of "eating a robot," as we did in this study, has great potential, as it may highlight subconscious human attitudes toward living organisms and robots, which cannot be quantified based on questionnaires. We believe that eating robots will be an attractive subject for stimulating interdisciplinary research discussions, and it is envisaged to be proven very important in developing discussions involving various researchers in the future."

**Comment 18**

In sum, I find this work highly interesting and innovative. I think it can contribute to various research fields including psychology and HRI and encourage a wide range of future studies. At the same time, I encourage the authors to clarify and justify some of the design and analysis choices, add a limitations section, and rewrite the discussion to better represent the profound impact of this interesting work.

Anderson-Bashan, L., Megidish, B., Erel, H., Wald, I., Hoffman, G., Zuckerman, O., & Grishko, A. (2018, August). The greeting machine: an abstract robotic object for opening encounters. In 2018 27th IEEE International Symposium on Robot and Human Interactive Communication (RO-MAN) (pp. 595-602). IEEE.

Erel, H., Shem Tov, T., Kessler, Y., & Zuckerman, O. (2019, May). Robots are always social: Robotic movements are automatically interpreted as social cues. In Extended abstracts of the 2019 CHI conference on human factors in computing systems (pp. 1-6).

Ju, W., & Takayama, L. (2009). Approachability: How people interpret automatic door movement as gesture. International Journal of Design, 3(2).

**Answer 18**

Thank you very much for your valuable suggestion. We have provided detailed information in response to your comment in our reply to Comment 1. In this response, we explained the rationale behind our design choices and addressed the limitations of our study. In addition, we have clarified and justified our design and analysis choices, added a limitations section, and rewritten the discussion to describe in detail the profound impact of our work. Furthermore, we have cited the three references you suggested as [26–28] in the Discussion section of our revised manuscript.

# Response to Reviewer 2

**General comment**

This study aimed to investigate the subjective response of eating an edible artifact. I have the following suggestions.

**Answer**

Thank you very much for your valuable comments. Response to all the comments are listed below.

**Comment 1**

The abstract should be improved by combining the objectives, short methodology, main findings result, and prospective application.

**Answer 1**

As you indicated, we have reviewed the abstract and rewritten it, addressing the objectives, short methodology, main findings, and prospective applications, as follows:

- Objectives: Investigated the effects of animated food consumption on human psychology
- Short methodology: Developed a pneumatically driven edible robot using gelatin and sugar, and evaluated participants' impressions associated with the visualization of the robot's movements and eating the robot
- Main findings: Participants perceived the moving robot to be more animate and felt guilty about consuming it; a difference in perceived texture when biting the robot under two conditions (moving and not moving) was observed
- Prospective applications: Valuable insights into the practical application of edible robots, such as in medical contexts

Revised abstract:
"This study aims to investigate the effects of consumption of animated food on human psychology. In this study, we developed a movable, edible robot and evaluated participants' impressions induced following the visualization of its movements and eating of the robot. While several types of edible robots have been developed, to our knowledge, no previous study has evaluated the effects associated with the eating of a robot. We developed a pneumatically driven edible robot using gelatin and sugar. Using this robot, we examined its perceived appearance and the participants' impression when it was eaten. In the robot-eating experiment, we evaluated two conditions: one in which the robot was moved and one in which it was not moved. Our results showed that participants perceived the moving robot to be more animated, and they felt guilty about consuming it. Additionally, we observed a difference in perceived texture when the robot was bitten under the two conditions. These findings provide valuable insights into the practical application of edible robots, such as in medical contexts."

**Comment 2**

The title is so fictitious and should be revised and align with novelty of this research.

**Answer 2**

Thank you very much for your suggestion. We totally agree to your comment and have updated the title to show the novelty of our research as below:

"Exploring the eating experience of a pneumatically driven edible robot: Animateness, guilt, and texture perception"

**Comment 3**

Please write down the contribution of the study at the end part of the Introduction section in bulleted form.

**Answer 3**

Thank you for your valuable suggestion. We have revised the Introduction section to explicitly state the contribution of our study in bulleted form at the end of the section, as follows:

> 5th paragraph, Introduction (page 2, line 40)
The contributions of this study are threefold:

- Development of a movable, edible robot intended for consumption. We designed and built a pneumatically driven edible robot using gelatin and sugar, incorporating flavor and a soft texture. This represents a novel approach, as previous edible robotics research [6–9] did not focus on the actual eating experience.
- Experiment 1. We evaluated participants' impressions of the robot solely based on the visualization of its movements, without actually eating it. This provided insights into how the perceived animateness of the robot might affect the hypothetical eating experience.
- Experiment 2. In this robot-eating experiment, by comparing two conditions, one in which the robot was moved and one in which it was not moved, we identified differences in participants' guilt about consuming the animated food and differences in perceived texture when biting the robot.

We believe that these contributions will be useful for readers to better understand the significance of our study.

**Comment 4**

Authors need to add human experimental protocol and study group size. Need to add IRB if available.

**Answer 4**

The human experimental protocol is described in the Procedure and Ethical considerations sections of each experiment. The group size is also indicated in the Participants section of each experiment. While not in the IRB format, these study protocols were approved by the Ethics Committee of the Graduate School of Engineering Science, Osaka University.

**Comment 5**

Authors only reported subjective results, may be influenced by bias. Objective results should be more appropriate.

**Answer 5**

Thank you for your valuable suggestion. Unfortunately, in this study, we did not conduct objective evaluations in this experiment. However, we completely agree that incorporating objective measures would be advantageous for such a novel study. For example, in Experiment 2, instead of relying solely on subjective questionnaires, we could have assessed participants' guilt or hesitation about eating the robot by measuring the time it took for them to actually bite and eat it after being instructed to do so. Since the robots are powered by air pressure, when bitten, the air pressure inside the air chamber decreases, which could be used to measure the time to bite. We would like to consider incorporating such objective evaluations based on behavior in future research. We have added a note about the lack of objective evaluations as limitations in the Discussion as follows:

> 4th paragprah, Discussion (page 17, line 517)
"In this study, we recognize the limitation of not incorporating objective evaluations, which could have provided additional insights into participants' guilt or hesitation about eating the robot. For instance, in Experiment 2, measuring the time it took for participants to bite and eat the robot after they were instructed to do so could have served as an objective measure that could complement the subjective questionnaires. As the robots are powered by air pressure, the decrease in air pressure inside the air chamber upon biting could be utilized to measure the time to bite. In future research, we aim to address this limitation by incorporating objective evaluations based on behavior, thus enhancing further the validity of our findings."

We acknowledge that the lack of objective measures, such as the time it takes for participants to bite and eat the robot, is a limitation of our study and we aim to address this in future research.
