# Supplementary material for: Exploring the eating experience of a pneumatically-driven edible robot: Perception, taste, and texture
Source: PLoS One. 2024 Feb 5;19(2):e0296697. doi: 10.1371/journal.pone.0296697 (PMC10843494; doi:10.1371/journal.pone.0296697)
Supplement: Attachment — Submitted filename: Response_to_Reviewers.pdf [file pone.0296697.s003.pdf]

Response to Reviewer 1

General comment

While many of my concerns were addressed, I have a few suggestions for the final version of the paper:

Answer

Thank you for your continued feedback and for highlighting additional suggestions for the previous version of our paper. We will answer each suggestion below.

Comment 1

1. Comment 2 - Clarify that the gestures were not designed to convey a specific intent or characteristic but rather to signal that the robot is capable of movement and acknowledge that participants may have assigned meaning to the movements that you did not evaluate

Answer 1

In the 1st round of peer review for this paper, we received the following comments:

Comment 2

"It is important to explain what was the predicted experience for each of the six videos and to translate the technical variables into experience terms. What did the authors expect participants to experience and why the specific technical manipulation of the object is predicted to lead to such experience."

We provided the following response:

"The six videos were designed to showcase six different movements achieved, through a combination of two motions (lateral swinging and vertical stretching), which can be manipulated by varying the timing of air supply to the two air chambers inside the edible part, as well as three levels of speed achieved by adjusting the cycle of air supply and exhaust. The lateral swinging motion produces movements similar to spinal flexion, while the vertical stretching motion produces movements similar to the up-and-down motion of the torso caused by knee flexion and extension. We considered that the speed of the movements could express the difference in alertness, where fast movements can appear exciting, and slow movements can appear relaxed. Although it cannot be claimed that faster movements are more lifelike, we expected that movements that are too slow would compromise the robot's lifelikeness."

The comment of this round is the following:

"Clarify that the gestures were not designed to convey a specific intent or characteristic but rather to signal that the robot is capable of movement and acknowledge that participants may have assigned meaning to the movements that you did not evaluate"

Therefore, a new description of Experiment 1 should be added:

> 1st paragraph, Robot movement conditions, Experiment 1 (page 8, line 248):

"Two factors were adjusted to control the movement of the developed edible robot: the timing and cycle of air supply and exhaust to and from the two air chambers in the edible part. For the timing of the air supply and exhaust, we set up two conditions: the first was one air chamber at a time (hereafter,

referred to as the "alternating" condition), and the second condition was the cycle of air supply and exhaust in which air was supplied to both air chambers (hereafter referred to as the "simultaneous" condition). In the alternating condition, the robot swings laterally, producing movements reminiscent of spinal flexion. By contrast, in the simultaneous condition, the robot moves up-and-down, generating movements akin to the up-and-down motion of the torso caused by knee flexion and extension. These movement patterns were selected based on the range of movements that the developed edible robot could realistically achieve, given its design and mechanism. Notably, terms such as "spinal flexion" or "movements of the torso" were selected as an illustrative comparison to enable readers to sufficiently understand the movement dynamics of the robot. The information regarding these motions was not disclosed to the participants, and we did not investigate their specific interpretations of these motions. Our primary objective was to investigate the psychological impact of various movements of the edible robot, rather than replicating human movement. In addition, for the air supply and exhaust cycle, we set up three conditions: short, middle, and long (see Table 4 for each condition). We considered that the speed of the movements could express the difference in alertness, with fast movements expressing excitement and slow movements expressing relaxation. Notably, fast movements are not inherently lifelike. However, we hypothesize that exceedingly slow motions might undermine the perception of the robot's animateness. Six conditions were set up as aforementioned, and the participants were involved in all conditions."

In addition, as limitations, we highlighted the possibility that the participant may have given a behavior a meaning that was not intended in the experiment.

> 1st paragraph, Limitations, Discussion (page 16, line 534):

"This study has the following limitations. The movements of the edible robot were not designed to convey a specific intent or characteristic but were determined based on what the developed robot could realistically achieve. However, participants might have attributed meanings to the movements of the robot that were not intended by us."

Comment 2

2. Comment 4 - add to the limitations that using these pre-tests may have primed these subjects in participants' minds, which could have biased their perception

Answer 2

Thank you for highlighting the concern regarding potential priming effects. We acknowledge the importance of this point and regret that we did not clearly specify in our initial manuscript that the participants in Experiments 1 and 2 were distinct groups. This means that no priming effects were influencing the participants' perceptions in Experiment 2 based on any previous exposure from Experiment 1.

For better clarity and to address your concern directly, we have incorporated the following statement in our manuscript:

> 1st paragraph, Participants, Experiment 2 (page 10, line 363):

"The experiment was conducted using students at Osaka University, Japan. The participants comprised 10 males and 6 females (mean age = 20.88 years old, range = 19–25 years old, and SD = 1.59 years old). Notably, the participants in Experiment 2 were distinct and did not overlap with those from

Experiment 1. All participants were Japanese. We specifically chose Japanese participants because of the cultural influences on the use of onomatopoeic terms, which will be elaborated in a subsequent section. Our major concern was whether cultural background could significantly affect the evaluation using onomatopoeia."

Comment 3

3. Comment 6 - I appreciate the authors' indication that the 2 subsets are independent; however, my comment concerned their analysis of single items, which is problematic. I suggest grouping the single items into 2 independent subscales and analyzing the 2 subscales instead of single items. By also adding Cronbach alpha analysis for each subscale, the authors will greatly improve the quality of their results.

Answer 3

Thank you for pointing this out. As you highlighted, our initial analysis focused on single items. However, in response to your feedback, we conducted factor analyses for both Experiments 1 and 2 and extracted two subscales from each experiment. We also ensured the quality of the results by calculating alpha coefficients.

Following your comments, we conducted a factor analysis and extracted two factors for both Experiments 1 and 2: "perception of the robot" and "taste." Consequently, these two factors were used as dependent variables instead of individual items. The results of this factor analysis have been included in the revised manuscript as new Tables 5 and 8 (Previous Table 5 has been removed). Moreover, using the scores derived from the factor analysis, we conducted an ANOVA for Experiment 1 and t-tests for Experiment 2. As a result, new Figs. 7 and 8 have been added.

We also added some sentences to the discussion section related to the results.

> 2nd paragraph, Discussion (page 16, line 506):

"The experiments investigated the influence of the movements of the edible robot on participants' impressions, particularly regarding their perception of the edible robot and its taste. In Experiment 1, we assessed the participants' impressions on different robot movements through an online survey. Our findings revealed that alternating movements were associated with higher perception and taste factor impression toward the edible robot than simultaneous movements. Additionally, middle- and short-cycle movements exhibited a higher perception impression of the edible robot than long-cycle movements. Therefore, in Experiment 2, we implemented the middle cycle of alternating movements in an edible robot and examined the impressions of the participants when consuming a moving robot. The results indicated that their perception of the edible robot was influenced by its movement, with the moving robot eliciting different impressions compared with the stationary robot. Moreover, a noticeable difference in the perceived texture emerged when participants bit and chewed on the robot under two distinct conditions. When biting and chewing a moving robot, they consistently reported experiencing specific textures (onomatopoeia): "Kori-Kori (Crisp)" and "Gabu (Grappling)." Conversely, when the robot was not in motion, the participants typically perceived a different texture (onomatopoeia): "Munya-Munya (Mumble)." Through this approach, we scrutinized the tactile sensations of eating in detail using onomatopoeia as a measure. Results revealed that despite being artificial, our robot movements could create different textures. Our study results suggest that the incorporation of specific movements into the robot can cause the participants to generate a high perceptions toward the robot. A possible interpretation is that when consuming the edible robot, the participants experienced heightened

perceptions because they attributed lifelike qualities such as emotion, intelligence, and animateness to the robot. The utilization of edible robots in this study enabled us to examine the effects of subtle movement variations in human eating behavior under controlled conditions, a task that would be challenging to accomplish with real organisms."

We would appreciate your confirmation of the results.

Table 5. Pattern matrix of Experiment 1

	Factor	
	1	2
**Perception** ($\alpha = 0.917$)		
Emotion	0.988	-0.048
Animateness	0.840	0.040
Intelligence	0.813	0.057
**Taste** ($\alpha = 0.925$)		
Do you think the object in the video looks tasty?	-0.005	0.954
Do you want to eat the object in the video?	0.032	0.888

Factor extraction method: Maximum Likelihood Method
Rotation method: Promax method with Kaiser's normalization

Table 8. Pattern matrix of Experiment 2

	Factor	
	1	2
**Perception** ($\alpha = 0.858$)		
Intelligence	0.921	-0.103
Emotion	0.898	-0.117
Animateness	0.662	0.070
Guilty	0.650	0.192
Freshness	0.588	0.052
**Taste** ($\alpha = 0.661$)		
Sourness	-0.163	0.842
Spiciness	0.040	0.737
Bitterness	0.211	0.708

Factor extraction method: Maximum Likelihood Method
Rotation method: Promax method with Kaiser's normalization

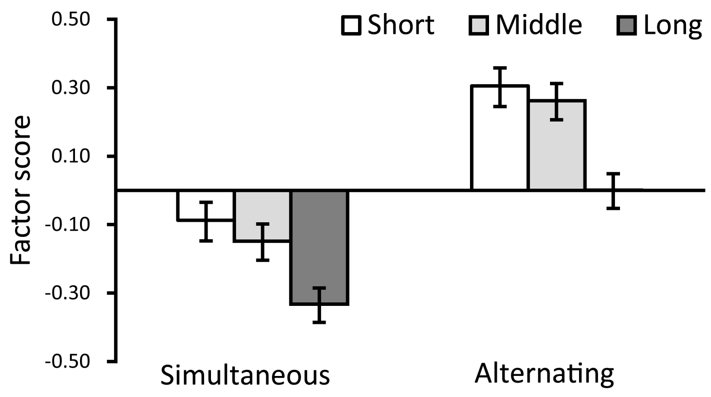

Fig 7. Results of Experiment 1: Factor 1 (perception).

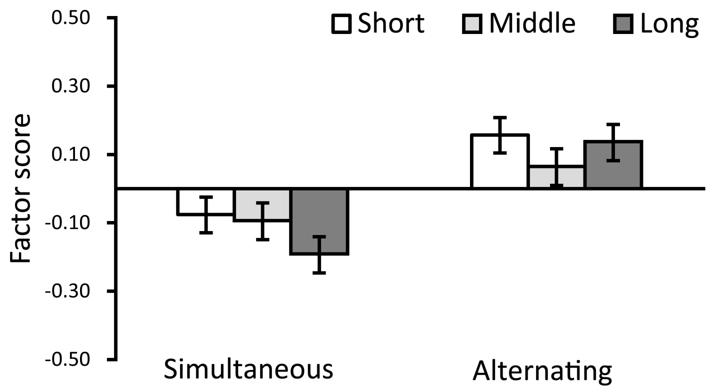

Fig 8. Results of Experiment 1: Factor 2 (taste).

Comment 4

4. Comment 7 - Without a clear analysis methodology, the qualitative data should be treated with extra caution. This should be clearly stated. In addition, percentages should be added to the word tables.

Answer 4

Thank you for pointing out the need for clarity in our qualitative analysis methodology. We agree that qualitative data should be treated with caution. To address your comment:

1. We added a note to the tables and included an explicit statement in the text.
2. Tables 6, 7, 9, 10, and 11 have been updated to present the results from our text-mining analysis, depicting the top five characteristic words for each condition. Based on your recommendation, we incorporated the Jaccard coefficient as an indicator to highlight the strength of association with each condition. This addition aids readers in understanding the overlap between word occurrences in different conditions.
3. We have cited the Jaccard coefficient using the provided references, enabling readers who are interested to explore the topic further.

We hope these adjustments address your concerns and enhance the clarity and robustness of our paper.

[Reference]
Osgood, C.E. (1959). The representational model and relevant research methods. In I. de S. Pool (Ed.), Trends in content analysis (pp. 33-88). University of Illinois Press.

Table 6. Free-form descriptions: What impression did you have of the object's movements?

Simultaneous	Jaccard coefficient	Alternating	Jaccard coefficient
Hard	0.068	Soft	0.079
Impression	0.057	Disgusting	0.055
Movement	0.055	Intense	0.054
Shaking	0.040	Not particularly	0.051
Stiff	0.036	Pulling	0.047

Note: Showing the top 5 words for each condition.

Table 7. Free-form descriptions: What do you think it tastes like?

Simultaneous	Jaccard coefficient	Alternating	Jaccard coefficient
Tasteless	0.090	Sweet	0.131
Orange	0.023	Jelly	0.056
Bitter	0.022	Delicious	0.026
Pasta	0.021	Gummi	0.023
Salty	0.020	Apple	0.023

Note: Showing the top 5 words for each condition.

Table 9. Free-form descriptions: What was your impression of what you just ate?

Stationary	Jaccard coefficient	Movement	Jaccard coefficient
Eating	0.250	Movement	0.242
None in particular	0.138	Creature	0.161
Food	0.129	Mouth	0.147
Jelly	0.103	Disgusting	0.125
Delicious	0.100	Bite	0.094

Note: Showing the top 5 words for each condition.

Table 10. Free-form descriptions: What did it taste or smell like?

Stationary	Jaccard coefficient	Movement	Jaccard coefficient
Smell	0.237	Sweet	0.161
Juice	0.100	None in particular	0.129
Eat	0.100	Apple	0.121
Fruit	0.094	Juice	0.069
Sweets	0.067	Flavor	0.069

Note: Showing the top 5 words for each condition.

Table 11. Free-form descriptions: What food ingredients do you think what you just ate is made of?

Stationary	Jaccard coefficient	Movement	Jaccard coefficient
Gelatin	0.419	Agar	0.231
Sugar	0.192	Fruit juice	0.217
Water	0.167	Sweetness	0.182
Apple	0.160	Artificial	0.095
Juice	0.130	Taste	0.095

Note: Showing the top 5 words for each condition.

Comment 5

5. Comment 8 - my comment did not relate to phrasing in general but to strong claims that are not supported by data (see my example in the original comment),

Answer 5

Thank you for your feedback. I have thoroughly reviewed the entire manuscript again and have made corrections to strong claims that were not supported by data.

For example, the abstract has been revised as follows:

> Abstract (page 1):

"This study investigated the effects of animated food consumption on human psychology. We developed a movable, edible robot and evaluated the participants' impressions induced by the visualization of its movements and eating of the robot. Although several types of edible robots have been developed, to the best of our knowledge, the psychological effects associated with the eating of a robot have not been investigated. We developed a pneumatically driven edible robot using gelatin and sugar. We examined its perceived appearance and the participants' impressions when it was eaten. In the robot-eating experiment, we evaluated two conditions: one in which the robot was moved and one in which it was stationary. Our results showed that participants perceived the moving robot differently from the stationary robot, leading to varied perceptions, when consuming it. Additionally, we observed a difference in perceived texture when the robot was bitten and chewed under the two conditions. These findings provide valuable insights into the practical applications of edible robots in various contexts, such as the medical field and culinary entertainment."

Comment 6

6. Comment 9 - Add to limitations the participants number

Answer 6

The following text was added to the Discussion section as a limitation:

> 6th paragraph, Limitations, Discussion (page 17, line 578):

"Additionally, we acknowledge the inherent challenges associated with the within-participants experimental design used in Experiment 2. This design can introduce order effects, even if conditions are counterbalanced. For instance, after experiencing a robot that moves, subsequent exposure to a robot that does not move might bias the participant to perceive it as "dead". We selected a within-participant design in Experiment 2 because of time constraints. Nevertheless, these biases might exist, and we believe that future studies would benefit from inter-participant comparisons to mitigate such order effects. Additionally, another significant limitation is the small number of participants, with only 16 participants in Experiment 2. In the analysis of Experiment 2, significant differences were observed, and with the power of the test yielding a high value, it is likely considered sufficient to detect differences between conditions. However, the limited sample size could impact the generalizability of our findings, possibly increasing the risk of type II errors. Future replications of this experiment should consider enlarging the sample size to ensure robust and reliable outcomes."

General comment 7

7. Comment 15 - Please use the term "trend" instead of "significant trend" - adding the word "significant" is misleading.

Answer 7

Thank you for pointing this out. To avoid any misunderstandings, I have checked the passages where "significant trend" is used and changed to simply "trend."

We have also added the data of the multiple comparison results.

> 4th paragraph, Results, Experiment 2 (page 14, line 475):

"Subsequently, we examined whether the texture of the edible robot when bitten and chewed differed between the tested conditions. For texture, the participants were asked to multiple select the corresponding onomatopoeia. Table 12 presents the onomatopoeia selected by the participants in each condition. In the stationary condition, a significant difference in the onomatopoeia type (Cochran's $Q(13) = 42.23$, $p < 0.001$) was observed. Adjusted multiple comparisons indicated that "Munya-Munya (mumble)" was selected significantly more often than Musha-Musha (Munching), Picha-Picha (Splashing), Bori-Bori (Munching), Gatsu-Gatsu (Gobbling), Gari-Gari (Crunchy), Kari-Kari (Crunchy), and Kusha-Kusha (Crunchy; $p < 0.05$ for all pairs). However, in the movement condition, a significant difference in the onomatopoeia type (Cochran's $Q(13) = 48.07$, $p < 0.001$) was observed. Adjusted multiple comparisons indicated that "Kori-Kori (Crisp)" and "Gabu (Grappling)" were selected trend of more often than Bori-Bori (Munching), Gatsu-Gatsu (Gobbling), Gari-Gari (Crunchy), Kari-Kari (Crunchy), and Kusha-Kusha (Crunchy; $p < 0.10$ for all pairs)."

Comment 8

8. Comment 16 - Do not treat this finding as an indication that there is no relationship between the variables - Bayesian statistics should be conducted in order to test the lack of effects.

Answer 8

We appreciate your insightful comment regarding the use of Bayesian statistics. We understand the potential pitfalls of solely relying on Pearson's product-moment correlation coefficient to conclude the lack of relationships between variables.

Our primary intent of using the correlation analysis was not to definitively state a non-relevance, but rather to explain our rationale for analyzing the multiple dependent variables in Experiment 2 separately for perception and taste. To further address your comment and provide a broader view of the underlying structures in our data, we conducted a factor analysis for the questions in Experiment 2. This analysis extracted two factors, namely "perception of the robot" and "taste (we have presented the results of our analysis in the text as Figure 8)." Given these findings, and the specific goals of our analysis, we believe a Bayesian correlation analysis may not be necessary in this context.

Response to Reviewer 3

General comment

I was asked to review the first revision of this manuscript. In doing so, I did not judge whether the revision adequately address the concerns of the first round of reviews.

Answer

Thanks to the reviewer's valuable comments. Detailed responses to all comments are listed below.

Comment 1

Introduction

The creation of the HERI research is described as follows:

"On the basis of these insights, we conceived the concept of human–edible robot 13
interaction (HERI) research, which explores a new relationship between humans and 14
robots by mimicking the consumption of living creatures with the use of edible robots 15
that allows for control and regulation of their appearance and movements."

In order to operate within the HERI framework, the investigators must demonstrate the two essential features of their experimental system:

1. That they can mimic the consumption of living creatures with edible robots.
2. That altering robots' appearance and movements will alter human behavior.

The investigators state their approach for doing so:

"... we developed edible robots based on considerations of their 27 taste and eating sensations and conducted experiments to investigate the psychological 28 and cognitive effects of the consumption of these moving robots on participants."

The investigators conducted two studies:

"In a preliminary study, we investigated the visual impression impacted by 33
the movement of the developed edible robot. In the main study, we conducted an 34
experiment with an edible robot under two operating conditions: with and without 35
robotic motion. After parts of the robot were consumed by participants, we evaluated 36
their responses as these pertained to the animateness, deliciousness (taste and texture), 37
appetite, and sense of guilt for eating parts of the robot. These factors are considered 38
essential evaluation items in HERI."

What is missing from the Introduction, and the paper as a whole, are an explicit set of hypotheses and the predictions which follow from them. Without that framing of the work, this is an exploratory study that is helping to develop and demonstrate the two essential features of the HERI system. Exploratory, developmental studies are important, particularly when developing a new experimental paradigm. Please clarify this.

Answer 1

Thank you for your detailed feedback and valuable comments on our manuscript.

We have introduced the concept of HERI research to explore a new relationship between humans and robots. This approach utilizes edible robots to mimic the consumption of living creatures. It is a preliminary exploration into the potential psychological and cognitive implications of consuming moving entities, especially in scenarios where experiments with real living beings might be infeasible or ethically problematic.

As you rightly pointed out, this study is exploratory and also preliminary, emphasizing the development and demonstration of HERI system, especially a moving edible robot. Consequently, we did not set explicit hypotheses or predictions, focusing instead on showcasing these foundational aspects of the HERI framework.

In the Introduction, we incorporated clarifications to explicitly state the exploratory nature of this study, emphasizing its aim to lay the groundwork for further studies on HERI.

We have made the following additions to the Introduction section:

> 2nd paragraph, Introduction (page 1, line 13):

"Because of the challenges of investigating consumption behaviors using living creatures, we introduced the concept of human–edible robot interaction (HERI). This study elucidates the interaction between humans and edible robots by offering a controlled environment to investigate human psychology when engaging with robots that are consumable even in motion. We recognize that the full replication of animality in edible forms remains a distant goal for HERI. Therefore, this study focuses on the immediate psychological responses elicited by the consumption of movable robots, rather than mimicking living organisms. Our primary aim in this initial phase is to investigate the psychological and cognitive effects that arise from such novel interactions."

> 4th paragraph, Introduction (page 2, line 32):

"Given the novelty of this research direction, our study primarily stands as exploratory, laying the foundational insights and understanding in the field of HERI. Herein, we examined the development of an edible robot whose moving part can be bitten and chewed and evaluated the participants' impressions on this act. First, we developed a pneumatically driven robot with a hardness suitable for biting and chewing using gelatin and sugar. In a preliminary study, we investigated the visual impression impacted by the movement of the developed edible robot. In the main study, we experimented with an edible robot under two operating conditions: without robot movement (stationary condition) and with robot movement (movement condition). After parts of the robot were consumed by participants, we evaluated their responses to the animateness, deliciousness (taste and texture), appetite, and sense of guilt for eating parts of the robot. These factors are considered essential evaluation items in HERI."

Comment 2

Edible Robot

The investigators designed and built an edible robot; to be appropriate for the HERI framework, the robot should address the first feature (stated above) needed for HERI. But it is not clear what design specifications the investigators were attempting to achieve. Why this design? From Figure 1, one can see the stick standing vertically. Given that HERI's goal is to explore the relationship between humans' eating behavior and edible robots, why, was this design created? Given the stated connection to humans eating live creatures, I was expecting a robot that mimicked a species that humans eat live or at least in a form that suggests to them the live, moving form. For example, worms are a common type of terrestrial and marine animal body form. Other researchers have built pneumatic worms, and the pneumatic stick would seem to be a better animal-like form if it were horizontal, on the table, compared to the stick wiggling in the air. It may be that a vertical stick mimics some live food; but please explain all of these design choices in light of the HERI objectives.

Answer 2

We express our gratitude for your interest in our research and your invaluable feedback.

The ultimate aim of HERI is to manufacture biologically-inspired, moving robots and to consume them. However, the primary objective of our study was to conduct an exploratory investigation into the psychological and cognitive reactions people have when consuming a moving robot. In this light, we initially developed manufacturing techniques for edible moving robots and explored the psychological and cognitive impacts when they are consumed.

This study focused on producing a robot that could be moved using edible materials. Therefore, we adopted pneumatics as a safe driving source for an edible robot. Additionally, we chose a stick shape as it offers a simple design that can be consumed while still in motion. Mimicking a specific biological form was a secondary objective at this stage.

In future studies aligned with HERI's direction, we intend to further emphasize the aspect of biological imitation.

We have made the following additions:

> 1st paragraph, Edible robot (page 2, line 59):

"This section describes the design, development, control method, and control system of the robot. The material used for the edible part of the robot and its characteristics were introduced. This study focused on manufacturing a robot that can be driven using edible materials. We chose a pneumatic approach for its drivability, given the harmlessness of air when ingested. Considering the actual eating while it moves, we designed the robot in a stick shape, a simple form. To conduct an experiment in which the edible part of the robot was eaten, materials were selected accordingly, and the robot was developed to satisfy the following conditions:

- Edible parts comprise materials that are safe to consume
- The edible part is not damaged when the robot moves but can be bitten and chewed as food"

> 2nd paragraph, Structure, Edible robot (page 3, line 81):

"Fig. 1 shows a three-dimensional (3-D) computer-aided design model of the edible robot and its internal structure. It consists of an edible part made of material will describe in the "Edible material" section and a base component, which is connected to an air tube to supply air to the edible part. As a pioneering endeavor in the field, we provided participants with the unique experience of consuming a robot while moving it. Thus, it was not feasible to cut the robot into smaller pieces before eating. Therefore, we had to design a shape and size that could enable the robot to be placed directly into the mouth. This led to stick-shaped robots. Additionally, owing to the constraint of the size of the robot that could fit in the mouth, the edible part was designed to have only two air chambers."

> 5th paragraph, Limitations, Discussion (page 17, line 569): :

"Our proposed edible robot design does not specifically mimic any particular biological form. To address these limitations, we will focus on the field by designing edible robots that imitate forms relevant to ongoing discussions on food shortages and cultural delicacies. Specifically, in future studies, we will emulate creatures consumed in contexts such as insect-based diets, which are being considered as a solution to food scarcity issues, and traditional Japanese dishes like "Odorigui" or "Ikizukuri (live fish sashimi)." These imitations are expected to provide deep insights into the psychological and cognitive responses elicited when consuming moving robots, merging technology with necessities and culinary traditions."

Comment 3

Edible material

Were taste tests conducted on the various materials? How, for example, do tensile stiffness and hardness relate to perceptions of flavor or tastiness? Were these parameters meant to be manipulated as part of the experimental design? If so, why? If not, then why were these mechanical tests included?

Answer 3

Thank you for the constructive feedback.

Regarding the “Edible Material” section:

Taste tests:

We did not conduct specific taste tests for the edible materials used in the robot. Our primary focus was to ensure that the edible parts of the robot were safe for consumption and met the physical requirements for operation. Taste was not a primary consideration during the selection or creation of the edible material.

Tensile stiffness, hardness, and taste:

We did not directly explore the relationship between tensile stiffness, hardness, and perceptions of flavor. Our mechanical tests ensured that the robot was functional during operation and chewable. The hardness measurements, for instance, were benchmarked against “Hard gummi” to ensure that the robot was edible.

Relation to experimental design:

Mechanical properties, such as tensile stiffness and hardness, were evaluated to test the robot’s functionality. The robot had to withstand pneumatic pressures without rupture and still be soft enough for participants to consume. These characteristics were vital for the design and feasibility of our study.

Purpose of mechanical tests:

These tests demonstrated that the edible parts of the robot met our design specifications, ensuring operability under pneumatic pressure and edibility.

We hope this clarifies the rationale behind our choices in material evaluation and design. We appreciate the opportunity to elaborate on these points.

We have made the following additions:

> 1st paragraph, Edible material, Edible robot (page 4, line 122):

“In the development of our pneumatically driven edible robot, one of the crucial challenges was to identify a material that enabled the necessary movement and inflation and was also edible and had a texture suitable for consumption. The material needed to have sufficient elasticity to withstand the stresses of pneumatic action without tearing, while also being chewable and edible as a food when consumed. This section describes our selection and reasoning for the edible materials used in the robot.”

> 1st paragraph, Tensile-strength testing, Edible robot (page 4, line 152):

"To ensure that the edible robot can function optimally without tearing during pneumatic action, we used tensile-strength material testing. This testing compares our selected edible material with existing materials in terms of their tensile strength, especially focusing on the effects of calcium carbonate addition. Furthermore, a reproduction of the gelatin-based-pneumatic-actuator material developed by Shintake et al. [7] was made, whereafter we measured its characteristics and compared the results with those of our robot. The material was prepared using gelatin:glycerol:water = 1:1:8 based on the results of previous studies and was dried at room temperature (25°C for 2 days). In total, we used three materials: reproduced gelatin and glycerol (1:1) (Gelatin/Glycerol (1:1)), gelatin and sucrose (sugar) with calcium carbonate (our material, Gelatin/Sucrose with $CaCO_3$), and gelatin with sucrose and without calcium carbonate (Gelatin/Sucrose without $CaCO_3$)."

> 1st paragraph, Hardness measurements, Edible robot (page 6, line 194):

"The texture of the edible robot should enable movement and render it palatable. The hardness of the material was measured to determine whether the assumed edible part was edible. The hardness of the material was compared with that of commercially available gummies and the reproduced pneumatic actuator using gelatin material developed by Shintake et al. [7]. We fabricated the reproduced material using the same procedure for the material used in the tensile-strength testing. Notably, the tensile-strength testing results showed that the reproduced material was softer than that proposed by Shintake et al. [7]. A durometer (C-type [20], KOBUNSHI KEIKI CO., LTD.), which was vertically pressed into the test specimen, was used to measure the Asker C hardness. Fig 3(a)–(d) show the test specimens of the developed edible part, the reproduction of the material developed by Shintake et al., and the two types of commercially available gummies (Lotte Fit's BIG gummy soft and hard types, LOTTE CO., LTD). The measurement was performed five times for each specimen, and the presented results are the averages of the measured values."

Comment 4

Experiment 1

For the robot movement conditions, why compare the motions of the small stick to humans? I don't think that this is intended, but that description makes me think that you are trying to mimic human motions. This question connects back to my questions about your design target: what, specifically, are you trying to mimic? As for how the vertical stick moves, you can simply say that in one mode it bends side-to-side and in the other it elongates and shortens.

When designing the speed and mode of the movements, here, again, is where reference to real animals that are eaten live – as mentioned in the Introduction – is important. As for the specific durations, why are those specific three levels chosen?

When the questions were first presented, it wasn't clear that the survey was based on an existing instrument. Then Takahashi et al. [21] was cited after the questions. This is the cited paper:

Takahashi H, Ban M, & Asada M. (2016). Semantic differential scale method can reveal multi-dimensional aspects of mind perception. Frontiers in Psychology. 7:1717.

The problem is that Takahashi et al. (2016) does not contain any survey questions. Instead, that paper cites Gray et al. (2007) and Takahashi et al. (2014), neither of which is cited in this current manuscript. More importantly, the Gray et al. (2007) and Takahashi et al. (2016) papers are about mind perception of a variety of entities, something quite different, I think, to the intention here.

I did more research to see whether I simply hadn't dug deep enough into the literature. To justify the questions in their questionnaire, Takahashi et al. (2014), cite this paper, which is also not cited in the current manuscript:

Kanda, T., Ishiguro, H., Ono, T., Imai, M., & Nakatsu, R. (2002). An evaluation on interaction between humans and an autonomous robot Robovie. Journal of Robotics Society of Japan, 20(3), 1e9.

I obtained a copy of this manuscript, which is in Japanese. Using ChatGTP, I translated portions of the paper. From that translation, I learned that the work is on a humanoid robot, investigating how movement of that robot impacts psychological reactions of humans interacting with it. This seems even less related to the current project than the work on mind perception.

I've been unable to find evidence that the questions in this manuscript have any basis in previous research. Unfortunately, this current survey lacks any basis for validity and reliability. Without validity and reliability, we don't know what the survey is actually measuring.

Answer 4

Thank you for your comprehensive feedback. The following is the response to each point:

Comparison of robot and human movements:

Notably, our description of the robot's movements using terms like "spinal flexion" and the motion resulting from "knee flexion and extension" was incorporated in response to feedback from another reviewer. The intent was to provide a familiar frame of reference to help readers easily understand the type of movements our robot exhibited. We did not intend to directly mimic human or any specific animal movements.

Considering the mechanics, the edible robot's movement was manipulated by the timing and cycle of air supply to its air chambers. The "alternating" condition caused the robot to swing laterally, reminiscent of spinal flexion, and the "simultaneous" condition produced an up-and-down motion, akin to the torso's movement from knee flexion and extension.

We understand that this clarification might have introduced some confusion and appreciate your pointing it out. We have further refined our explanation in the revised manuscript to ensure clarity and prevent potential misunderstandings.

Reference to actual animal movements:

The HERI research primarily focused on investigating the psychological reactions to observing and consuming moving entities, especially edible robots. We acknowledge the potential associations made between the HERI research and the act of consuming live organisms. However, in this preliminary study within HERI research, our objective is not to simulate the exact movements or behaviors of any specific living creature. Instead, we aim to capture a range of psychological reactions to various robotic movements in a consumption context, without attempting to directly replicate the nuances of natural animal behaviors.

Our initial description might have unintentionally introduced ambiguity, and we sincerely apologize for any confusion. We have improve our explanation in the revised manuscript to sufficiently delineate our intent.

We have revised main text as follows:

> 1st paragraph, Robot movement conditions, Experiment 1 (page 8, line 248):

"Two factors were adjusted to control the movement of the developed edible robot: the timing and cycle of air supply and exhaust to and from the two air chambers in the edible part. For the timing of the air supply and exhaust, we set up two conditions: the first was one air chamber at a time (hereafter, referred to as the "alternating" condition), and the second condition was the cycle of air supply and exhaust in which air was supplied to both air chambers (hereafter referred to as the "simultaneous" condition). In the alternating condition, the robot swings laterally, producing movements reminiscent of spinal flexion. By contrast, in the simultaneous condition, the robot moves up-and-down, generating movements akin to the up-and-down motion of the torso caused by knee flexion and extension. These movement patterns were selected based on the range of movements that the developed edible robot could realistically achieve, given its design and mechanism. Notably, terms such as "spinal flexion" or "movements of the torso" were selected as an illustrative comparison to enable readers to sufficiently understand the movement dynamics of the robot. The information regarding these motions was not disclosed to the participants, and we did not investigate their specific interpretations of these motions. Our primary objective was to investigate the psychological impact of various movements of the edible robot, rather than replicating human movement. In addition, for the air supply and exhaust cycle, we set up three conditions: short, middle, and long (see Table 4 for each condition). We considered that the speed of the movements could express the difference in alertness, with fast movements expressing excitement and slow movements expressing relaxation. Notably, fast movements are not inherently lifelike. However, we hypothesize that exceedingly slow motions might undermine the perception of the robot's animateness. Six conditions were set up as aforementioned, and the participants were involved in all conditions."

Relevance of literature:

We acknowledge the thematic influence of Takahashi et al. [21] on our methodology, serving as a source of inspiration for our research. Although our questions were uniquely devised to align with the specific contours of our study, drawing on broader perceptual themes found in existing studies, we recognize the need for further refinement and rigorous validation of this questionnaire in future studies.

Ensuring the reliability and accuracy of this instrument in teasing apart these sophisticated constructs remains a paramount consideration.

Reliability and validity of the survey:

We understand the gravity of your concern regarding the lack of direct evidence for the validity and reliability of our survey. Given the novelty of the evaluation of edible robot impression, established tools could not be directly borrowed. We acknowledge the initial exploratory nature of our approach and the need for rigorous validation in future studies. This study serves as a foundational step, and we anticipate refining our tools as the research field matures.

We appreciate your detailed feedback, which will help improve the clarity and rigor of our work. We have implemented the necessary revisions.

We have revised main text as follows:

> 1st paragraph, Question items, Experiment 1 (page 8, line 273):

"To investigate perception and taste change only by watching the movements of the edible robot, participants were asked to respond to some items. For each of the six videos, the participants were asked to respond to the following eight items based on a seven-point Likert scale. We adopted the Likert scale methodology to quantitatively assess shifts in specific impressions and emotions. The following are the questionnaire items.

- Do you feel the object's animateness in the video?
- Do you think the object in the video has emotions?
- Do you think the object in the video has intelligence?
- Do you think the object in the video has a hard texture?
- Do you think the object in the video is fresh?
- Do you think the object in the video looks tasty?
- Do you want to eat the object in the video?
- Do you think eating the object in the video is against the moral code?

We used the term "object" instead of "edible robot" in the questions because the term "edible robots" could introduce a bias in the participants' answers. Notably, although our methodology was thematically influenced by Takahashi et al. [21], it did not involve a verbatim replication of their survey items. Our questions were uniquely devised to suit the specific contours of our research, drawing on broad perceptual themes elucidated in previous works. We acknowledge the imperative for further refinement and rigorous validation of this questionnaire in future research, ensuring its reliability and accuracy in teasing apart these sophisticated constructs. In addition to the eight separate Likert-scale questions, we asked the respondents to write freely about two other items: "What impression did you have of the object's movements?" and "What do you think it tastes like?" "

Comment 5

Experiment 2

Many of the same problems for Experiment 1's survey instrument are in play for Experiment 2's modified version. While I'm intrigued by the use of onomatopoetic terms in describing illness and food impressions, we still have the problem validity and reliability in the current context.

When considering the experimental design, it appears that all participants participated in stationary and animated conditions. What was the nature of the animated condition? Bending or elongation and shortening?

Of more concern is the lack of a set of controls to understand the effects of a vertical stick connected to a cup-like holder relative to the stick unattached that one picks up with one's fingers. A detached stick would also allow investigation of cutting and utensil use on perception. With controls, one could also seek to understand the effect of having to hold the food in the mouth for 10 seconds compared to just biting, the usual means of eating.

In terms of the results, it seems clear that the two different conditions – stationary or moving – impacted verbal impressions that people gave of what they ate. This is tentative support for the second of the two essential features of the HERI experimental system:

1. That they can mimic the consumption of living creatures with edible robots.
2. That altering robots' appearance and movements will alter human behavior.

However, the first feature is not addressed in this work.

Answer 5 Thank you for your valuable comments.

Reliability and validity of the survey:
We acknowledge your concerns about the validity and reliability of our survey. Given the lack of established tools for evaluating impressions on edible robots, we used direct questions. We understand this approach has its limitations. Your feedback is invaluable, and we will refine our methods in future iterations based on your insights.

Use of onomatopoetic terms:
Thank you for recognizing the intriguing approach we adopted using onomatopoetic terms. Because of the cultural influence on the use of these terms, especially in describing impressions, we believed that a consistent cultural background among participants was necessary. Thus, for this study, we ensured that all participants were Japanese. This choice ensured a homogenous cultural context for the interpretation of the onomatopoetic terms.

Concerns about the experimental design:
As detailed in our manuscript, the participants were subjected to two conditions: stationary and movement. For clarity, "The movement condition utilized was the middle cycle of the alternating condition, as mentioned in the AM conditions in Table 4."

Lack of a control set:
The design of the robot, specifically the necessity of the base component covered by the cup-like holder for supplying air for its movement, fundamentally influenced the participants' interaction with it. The edible part of the robot was attached to the base component, which was housed within the cup-like holder. Without the base component, the robot could not move, establishing a direct comparison with a

detached stick (edible part) infeasible in this study's design. Notably, the holder was used consistently across all conditions, stationary or moving.consistently across all conditions, whether the robot was stationary or moving.

This study primarily investigated the perceptual differences between moving and stationary robots. The 10-second-holding period in the mouth was designed to allow participants to experience the robot's movement, which would have been lost if they were to bite it immediately. Although we understand and appreciate the reviewer's suggestion to consider the influence of different consumption methods, such as immediate biting, this was beyond the scope of our study. However, it indeed presents a valuable avenue for future studies. We acknowledge this limitation, and future studies will aim to explore the effects of different consumption methods on perception.

Feedback on the results:
The aspect of "mimicking the consumption of living creatures with edible robots" is a long-term goal, and we did not assume it to be addressed in this study. Our primary focus was to scrutinize the cognitive and psychological effects when consuming movable objects.

In this light, we have revised the following sections of the manuscript:

> Abstract (page 1):

"This study investigated the effects of animated food consumption on human psychology. We developed a movable, edible robot and evaluated the participants' impressions induced by the visualization of its movements and eating of the robot. Although several types of edible robots have been developed, to the best of our knowledge, the psychological effects associated with the eating of a robot have not been investigated. We developed a pneumatically driven edible robot using gelatin and sugar. We examined its perceived appearance and the participants' impressions when it was eaten. In the robot-eating experiment, we evaluated two conditions: one in which the robot was moved and one in which it was stationary. Our results showed that participants perceived the moving robot differently from the stationary robot, leading to varied perceptions, when consuming it. Additionally, we observed a difference in perceived texture when the robot was bitten and chewed under the two conditions. These findings provide valuable insights into the practical applications of edible robots in various contexts, such as the medical field and culinary entertainment."

> 2nd paragraph, Introduction (page 1, line 13):

"Because of the challenges of investigating consumption behaviors using living creatures, we introduced the concept of human–edible robot interaction (HERI). This study elucidates the interaction between humans and edible robots by offering a controlled environment to investigate human psychology when engaging with robots that are consumable even in motion. We recognize that the full replication of animality in edible forms remains a distant goal for HERI. Therefore, this study focuses on the immediate psychological responses elicited by the consumption of movable robots, rather than mimicking living organisms. Our primary aim in this initial phase is to investigate the psychological and cognitive effects that arise from such novel interactions."

> 1st paragraph, Participants, Experiment 2 (page 10, line 363):

"The experiment was conducted using students at Osaka University, Japan. The participants comprised 10 males and 6 females (mean age = 20.88 years old, range = 19–25 years old, and SD = 1.59 years old). Notably, the participants in Experiment 2 were distinct and did not overlap with those from Experiment 1. All participants were Japanese. We specifically chose Japanese participants because of the cultural influences on the use of onomatopoeic terms, which will be elaborated in a subsequent

section. Our major concern was whether cultural background could significantly affect the evaluation using onomatopoeia."

> 1st paragraph, Question items, Experiment 2 (page 11, line 376):

"To examine whether impressions such as "perception" and "taste" for eating the edible robot changed in response to the robot's movements, the participants were asked to answer the following 13 Likert-scale question items for all stationary and movement conditions. They were asked to the following 13 items (some items are the same as in Experiment 1) using the seven-point Likert scale for each of the stationary and movement conditions. We adopted the Likert scale methodology to quantitatively assess shifts in specific impressions and emotions. Incidentally, in Experiment 1, we used the index of "morality," but in Experiment 2, we revised it to "guilt," a question item that is likely to be linked to a specific action, to evaluate the impression of eating a robot."

> 4th paragraph, Limitations, Discussion (page 16, line 552):

"Although the edible component is a pivotal tool for this study, it lacked autonomy in its functionality. For the robot to move, it should be connected to the base component for air supply. This design prerequisite restricted our presentation options for consumption. Specifically, serving only the edible part on a plate without its essential base was impractical. Considering the unwieldy nature of the base component, we adopted a practical solution by encasing it within a cup-like holder equipped with a handle for easier manipulation. However, this adaptation inadvertently introduces an uncertainty, the potential influence of the holder on the participants' perception of the edible robot. The current experimental design could not account for or assess the potential perceptual implications cause by the holder. Moreover, the study design required that participants should hold the robot in their mouth for 10 s to experience its movement. Although this was vital for our primary objective to compare the perceptions between a moving and stationary robot, it did not permit other consumption methods, such as immediate biting, which might elicit different perceptions. In future studies, we will investigate alternative consumption methods and their effects on perception. Furthermore, we will develop the edible parts of the edible robot to operate independently from the other components."

Response to Reviewer 4

General comment

The authors have addressed all the comments from the previous round. Therefore, I am happy to recommend acceptance at this stage.

Answer

Thank you for your positive feedback and recommendation. We deeply appreciate your time and insights throughout the review process.
